# Development of cellobiose-degrading ability in *Yarrowia lipolytica* strain by overexpression of endogenous genes

**DOI:** 10.1186/s13068-015-0289-9

**Published:** 2015-08-04

**Authors:** Zhongpeng Guo, Sophie Duquesne, Sophie Bozonnet, Gianluca Cioci, Jean-Marc Nicaud, Alain Marty, Michael Joseph O’Donohue

**Affiliations:** LISBP-Biocatalysis Group, INSA/INRA UMR 792, Université de Toulouse, 135 Avenue de Rangueil, 31077 Toulouse, France; INRA, UMR792 Ingénierie des Systèmes Biologiques et des Procédés, 31400 Toulouse, France; CNRS, UMR5504, 31400 Toulouse, France; INRA, UMR1319 Micalis, 78352 Jouy-en-Josas, France; AgroParisTech, UMR Micalis, 78352 Jouy-en-Josas, France

**Keywords:** Lignocellulosic biomass, Oleaginous yeast, Lipids, Enzymatic hydrolysis, Cellulases

## Abstract

**Background:**

*Yarrowia lipolytica*, one of the most widely studied “nonconventional” oleaginous yeast species, is unable to grow on cellobiose. Engineering cellobiose-degrading ability into this yeast is a vital step towards the development of cellulolytic biocatalysts suitable for consolidated bioprocessing.

**Results:**

In the present work, we identified six genes encoding putative β-glucosidases in the *Y. lipolytica* genome. To study these, homologous expression was attempted in *Y. lipolytica* JMY1212 Zeta. Two strains overexpressing *BGL1* (YALI0F16027g) and *BGL2* (YALI0B14289g) produced β-glucosidase activity and were able to degrade cellobiose, while the other four did not display any detectable activity. The two active β-glucosidases, one of which was mainly cell-associated while the other was present in the extracellular medium, were purified and characterized. The two Bgls were most active at 40–45°C and pH 4.0–4.5, and exhibited hydrolytic activity on various β-glycoside substrates. Specifically, Bgl1 displayed 12.5-fold higher catalytic efficiency on cellobiose than Bgl2. Significantly, in experiments where cellobiose or cellulose (performed in the presence of a β-glucosidase-deficient commercial cellulase cocktail produced by *Trichoderma reseei*) was used as carbon source for aerobic cultivation, *Y. lipolytica* ∆*pox* co-expressing *BGL1* and *BGL2* grew better than the *Y. lipolytica* strains expressing single *BGLs*. The specific growth rate and biomass yield of *Y. lipolytica* JMY1212 co-expressing *BGL1* and *BGL2* were 0.15 h^−1^ and 0.50 g-DCW/g-cellobiose, respectively, similar to that of the control grown on glucose.

**Conclusions:**

We conclude that the bi-functional *Y. lipolytica* developed in the current study represents a vital step towards the creation of a cellulolytic yeast strain that can be used for lipid production from lignocellulosic biomass. When used in combination with commercial cellulolytic cocktails, this strain will no doubt reduce enzyme requirements and thus costs.

**Electronic supplementary material:**

The online version of this article (doi:10.1186/s13068-015-0289-9) contains supplementary material, which is available to authorized users.

## Background

It is widely recognized that lignocellulosic biomass (or LC biomass) will form an important part of the future bio-economy. However, the use of this renewable resource as feedstock for industrial activities poses a major challenge, because its deconstruction to sugars and lignin is complex, requiring a series of unit operations. These include costly pretreatment and enzymatic hydrolysis steps, the latter requiring the action of several types of enzymes [1, 2]. Indeed, the hydrolysis of cellulose alone requires the synergistic action of endoglucanases (EC 3.2.1.4), cellobiohydrolases (EC 3.2.1.91) and β-glucosidases (EC 3.2.1.21) [3]. Endoglucanases are active on the internal bonds in cellulose and release free reducing and non-reducing extremities, which are used by cellobiohydrolases as starting points for exo-processive hydrolysis that yields cellodextrins as products. Finally, β-glucosidases convert cellodextrins into glucose [4].

One strategy to reduce investment and operational costs in LC biomass processing is to internalize enzyme production and combine enzymatic hydrolysis with fermentation. This is known as consolidated bioprocessing (CBP) and can be achieved using a microorganism that possesses the dual ability to produce biomass-hydrolyzing enzymes and ferment sugars to products of commercial interest, thus allowing a one-pot type bioconversion process in which process integration is maximized [5]. While CBP is considered to be an ultimate aim for biorefining, the ways to achieve this goal are not simple. Although the number of naturally occurring, biomass-degrading microorganisms is no doubt large, those that possess the ability to hydrolyze LC biomass and ferment free sugars into target products, such as ethanol, butanol, hydrogen, fatty acid ethyl esters (FAEE) or isopropanol, at industrially compatible rates and titers, are probably very rare and so far undiscovered [6]. Additionally, many of the best known biomass-degrading microorganisms display low β-glucosidase (cellobiase) activity, meaning that the hydrolysis of cellobiose constitutes a rate-limiting step during the enzymatic processing of cellulose [7–9]. Therefore, engineering cellobiose-degrading ability into microorganisms is a vital step towards the development of cellulolytic biocatalysts suitable for CBP. In this respect, examples of recent work performed on *Saccharomyces cerevisiae*, the current workhorse of biotechnological processes, are noteworthy [10–12]. In these studies, even though the engineered *S. cerevisiae* strains exhibited poor cellulose-degrading ability, the fact that they both produce significant cellobiase activity means that their incorporation into a simultaneous saccharification and fermentation (SSF) process is likely to reduce the loading of external cellulases and thus overall process cost [10].

Although ethanol is the target molecule in many biorefinery concepts, Fatty Acid Esters (FAEs) such as those used in biodiesel, are also attractive targets. This is because FAEs display high energy density and are well-tolerated by production strains [13]. Currently, FAEs are mainly produced by transesterification of plant oils using an alcohol (methanol or ethanol) and base, acid or enzyme catalysts [14]. However, the high cost of this process and various issues surrounding the production of plant oils for non-food purposes make the search for alternative routes both attractive and strategically pertinent. In this respect, microbial production of biofuels (so-called microdiesel and microkerosene) represents a sustainable and quite economical way to produce FAEs. For this purpose, both *Escherichia coli* and *S. cerevisiae* have been engineered to produce structurally tailored fatty esters [15–17]. However, neither of these microorganisms is naturally able to accumulate high amounts of lipids, nor able to degrade cellulose. Moreover, in these microorganisms the biosynthesis of fatty acid is highly regulated [18], thus limiting the possibility to improve lipid production [16, 17, 19].

So-called oleaginous microorganisms, which naturally accumulate lipids to more than 20% of their dry cell weight (DCW) [20, 21], have already been exploited for the production of commercially useful lipids, such as substitutes for cocoa butter and polyunsaturated fatty acids [22]. Therefore, it is unsurprising that microbial lipid or single cell oil is also being considered for biodiesel production, especially because this route implies shorter production times, reduced labor costs and simpler scale-up [23]. Prominent among the oleaginous microorganisms, *Yarrowia lipolytica* has been extensively studied and is known to accumulate lipids up to 50% of its dry weight depending on culture conditions [20, 21, 24]. Advantageously, since *Y. lipolytica* is already widely used in the detergent, food, pharmaceutical and environmental industries it has been classified by the FDA (Food and Drug Administration) as “Generally Recognized as Safe” (GRAS) for numerous processes [25]. Nevertheless, despite these advantages, *Y. lipolytica* displays limited ability for sugar use and is unable to use cellulose as carbon source [26].

**Fig. 1:**
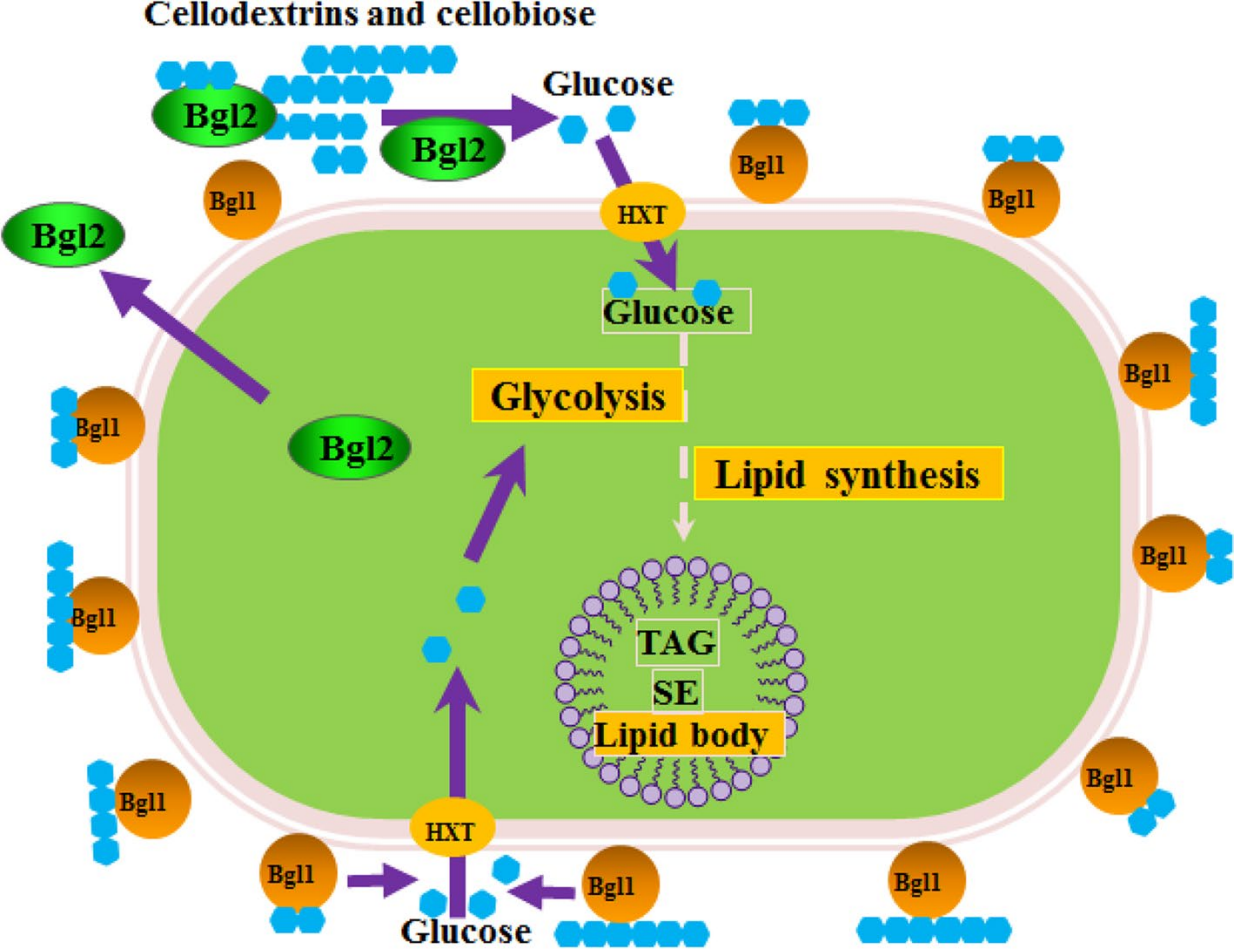
The strategies used in the current study to develop the cellobiose-degrading ability in *Y*. *lipolytica.*

In a recent paper, the use of cellobiose by *Y. lipolytica* was tackled for the first time, thus opening the way towards the development of an efficient yeast-based CBP microorganism capable of consuming cellulose-derived glucose and converting it into lipids and derivatives thereof [27]. Herein, we present work that shares this aim, but which has employed a different strategy that relies upon the activation of endogenous β-glucosidase activity (Fig. 1).

## Results

### Identification of genes encoding active β-glucosidases in *Y. lipolytica*

**Fig. 2:**
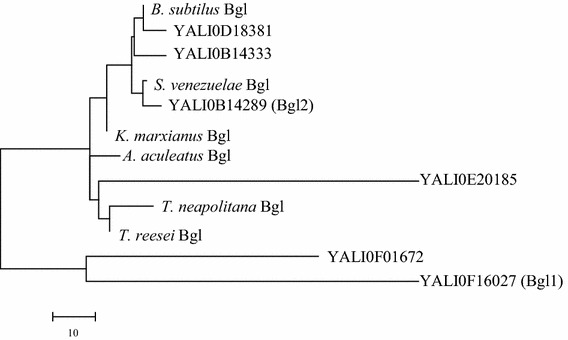
Phylogenetic tree of the structurally characterized family three glycosyl hydrolases. Multiple-sequence alignment was performed using CLUSTALW (http://www.genome.jp/tools/clustalw/), and a phylogenetic tree was constructed using MEGA5 (http://www.megasoftware.net/). Sequences are: *Bacillus subtilus* Bgl (PDB accession number 4GYJ_A); *Streptomyces venezuelae* Bgl (PDB accession number 4I3G_A); *Kluyveromyces marxianus* Bgl (PDB accession number 3AC0_A); *Aspergillus aculeatus* Bgl (PDB accession number 4IIB_A); *Thermotoga neapolitana* Bgl (PDB accession number 2X42_A); *Trichoderma reesei* Bgl (PDB accession number 4I8D_A); and include the 6 putative GH3 sequences from *Yarrowia lipolytica* genome.

Analysis of the *Y. lipolytica* genome using BLAST revealed the presence of six sequences that were identified as putative family GH3 β-glucosidases (see Additional file 1: Table S1; Additional file 2: Fig. S1) on the basis of high amino acid sequence identity with other yeast β-glucosidases (Fig. 2, Additional file 2: Fig. S1). However, in the absence of biochemical data it was impossible to assert at this stage that these sequences actually encode β-glucosidases, since family GH3 contains glycoside hydrolases that display other specificities. Moreover, *Y. lipolytica* does not grow on cellobiose and has not been found to express a detectable level of β-glucosidase activity (Additional file 2: Fig. S2), despite the fact that preliminary transcriptional analysis revealed that the six genes are weakly transcribed when *Y. lipolytica* is grown on glucose, although no further induction was observed in the presence of cellobiose (Additional file 2: Fig. S3). In this respect, it was particularly gratifying to observe that overexpression of *BGL1* (YALI0F16027g) or *BGL2* (YALI0B14289g) in *Y. lipolytica* (strains ZetaB 1 and ZetaB 2, respectively) enhanced the transcription of the genes and conferred the ability to grow on solid medium containing cellobiose as the sole carbon source (Additional file 2: Figs. S2, 3). Additionally, when these recombinant strains were grown on YNB-*p*NP-βGlc (*p*-nitrophenyl-β-d-glucoside) plates, yellow halos surrounding the colonies were clearly visualized, indicating β-glucosidase activity (Additional file 2: Fig. S3). Finally, after growth in liquid YTD medium, β-glucosidase activity could be measured in the cell extract of ZetaB 1 (3.2 ± 0.2 IU/mg) and in the culture supernatant of ZetaB 2 (2.6 ± 0.1 U/mL), while much lower activities were measured in the culture supernatant of ZetaB 1 (0.33 ± 0.02 U/mL) and in the cell extract of ZetaB 2 (0.42 ± 0.01 IU/mg). Regarding the remaining four putative β-glucosidases, expression of their encoding sequences (YALI0F01672g, YALI0D18381g, YALI0B14333g and YALI0E20185g) in *Y. lipolytica* failed to produce any detectable β-glucosidase activity or sustain yeast growth on solid medium containing cellobiose as the sole carbon source.

**Fig. 3:**
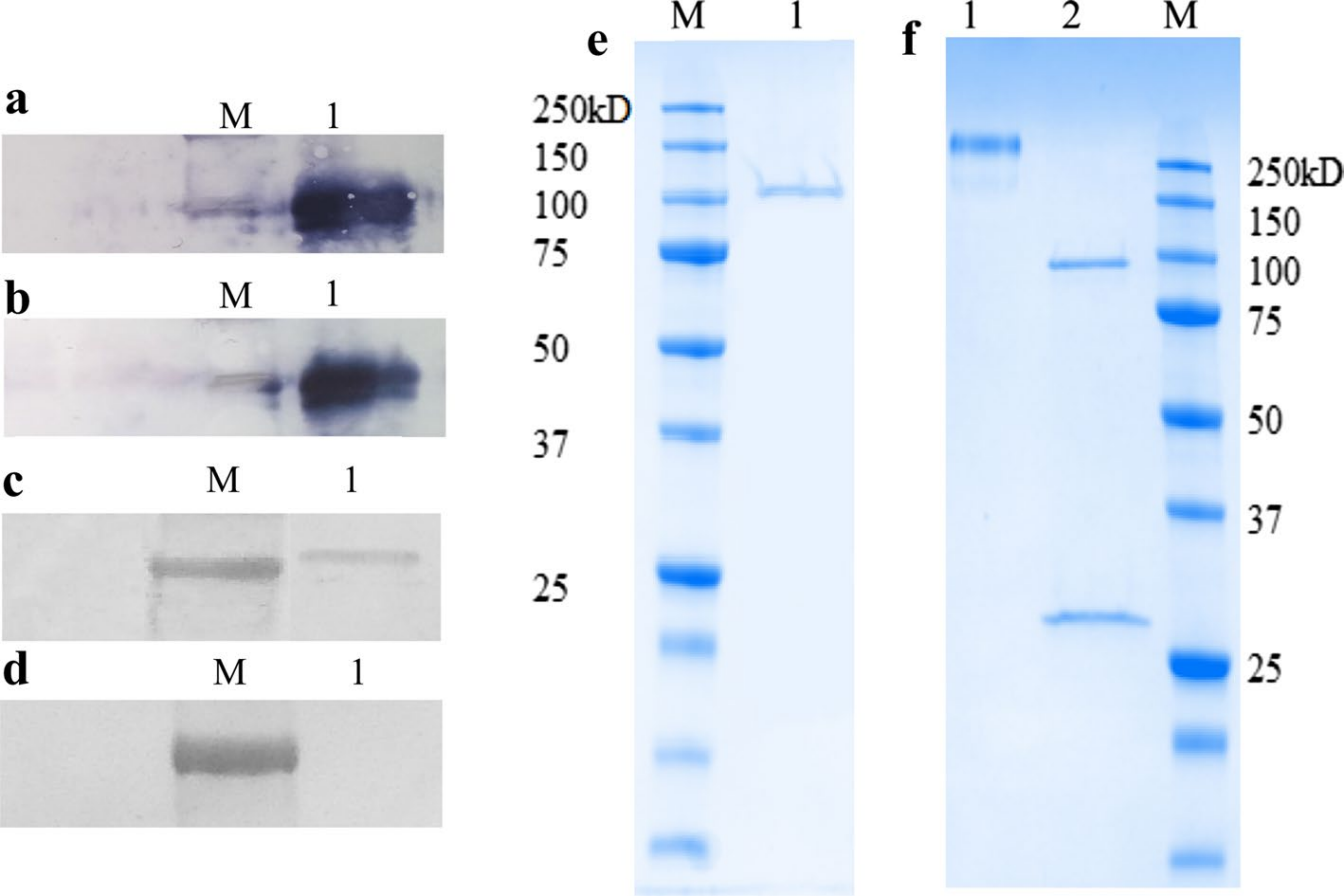
Western blot detection of the expressed β-glucosidases **a** M, molecular weight standards; *lane 1*, intracellular Bgl1, **b** *lane 1*, extracellular Bgl1-His6, **c** *lane 1*, intracellular Bgl2-His6, **d** *lane 1*, extracellular Bgl2-His6, and SDS-PAGE analysis of the purified β-glucosidases from *Y. lipolytica* JMY1212 transformants **e** *lane 1*, purified Bgl1-His6, and **f** *lane 1*, purified Bgl2; lane 2, endo-H treated Bgl2 (The *lower band* in *lane 2* represents the expected size of Endo-H).

To further investigate the production of the six β-glucosidases, western blot analysis was carried out using anti-His6 antibodies. This revealed the presence of Bgl1-His6 in both the culture supernatant and cell extract (Fig. 3a, b). Bgl2-His6 could only be detected in concentrated (tenfold) cell extract (Fig. 3c). However, this method failed to detect Bgl2-His6 in the culture media (Fig. 3d), consistent with the fact that no β-glucosidase activity was detected in this fraction. Significantly, the expression of the native *BGL2* sequence (i.e. without the His6-tag) provided much more satisfactory expression, implying that the presence of the His6-tag on Bgl2 somehow impairs the expression and/or secretion of this protein.

### Localization of β-glucosidases in ZetaB 1 and ZetaB 2

To determine the localization of Bgl1 and Bgl2, yeast cells producing these enzymes (without His6 tag) were fractionated, generating on the one hand extracellular samples (culture supernatant), and on the other cell-associated periplasmic, cytoplasmic and membrane fractions. Measurement of the β-glucosidase activities in each of these fractions revealed that Bgl1 was primarily localized in the periplasm (61%), but was also present in the cytoplasm (30%), while Bgl2 was mainly in the supernatant (80%), and to a lesser extent (26%) in the periplasm (Table 1). The apparent ambiguity of these results is undoubtedly due to the separation method that was employed to isolate the different cellular fractions, which inevitably led to a low level of cross-contamination between the samples. Nevertheless, taking this into account, it is reasonable to deduce that Bgl1 is primarily localized in the periplasmic space, while Bgl2 is secreted into the culture medium.

**Table 1:** Distribution of β-glucosidase activity in recombinant strains ZetaB 1 and ZetaB 2

Fraction	Relative enzyme activity^a^	
	Bgl1 (%)	Bgl2 (%)
Total	100	100
Growth medium	2.3 ± 0.4	79.6 ± 1.2
Periplasm	60.7 ± 1.0	25.8 ± 1.0
Cytoplasm	30.0 ± 0.5	4.8 ± 0.8
Membrane	8.1 ± 0.7	3.7 ± 0.3

±, the standard deviation.

^a^Triplicate experiments. Activity was assayed with pNP-βGlc.

### Production, purification and characterization of Bgl1 and Bgl2

Production of Bgl1-His6 and native Bgl2 was achieved by growing the appropriate *Y. lipolytica* strains on YTD, with expression of both enzymes increasing until complete depletion of glucose was reached (36 h).

Regarding purification of Bgl1-His6, yeast cells from a 200-mL culture volume yielded approximately 550 U of enzyme in the crude cell extract. However, after one step of affinity-purification, only 17% of Bgl1 was recovered (Table 2). In the case of Bgl2, a two-step protocol using anion exchange chromatography and gel filtration allowed its purification to near homogeneity, but led to significant loss of protein (8.8% recovery). SDS-PAGE analysis of the two purified protein samples revealed that while the *M*_r_ of Bgl1 was consistent with the expected value (i.e. *M*_r_ of 90.4 kDa for the protein lacking the putative signal peptide), that of Bgl2 was significantly higher (>250 kDa) (Fig. 3e, f). To investigate whether this discrepancy was due to glycosylation, the amino acid sequence of Bgl2 was analyzed using the glycosylation predictor GlycoEP (http://www.imtech.res.in/raghava/glycoep/) [28]. This revealed that Bgl2 harbors 18 potential N-glycosylation sites (Additional file 1: Table S2). Glycosylation was finally confirmed by treating the purified Bgl2 protein with endoglycosidase H (EndoH) and migrating it on a SDS-PAGE. This analysis revealed that the *M*_r_ of the recombinant EndoH-treated Bgl2 was approximately 95 kDa, quite consistent with the theoretical *Mr* of 92.9 kDa (Fig. 3f). Finally, N-terminal amino acid sequence analysis of Bgl1 and Bgl2 confirmed that the signal sequences of both proteins had been cleaved and allowed the accurate localization of the cleavage sites (Additional file 2: Fig. S4).

**Table 2:** Purification of intracellular Bgl1-His6 and extracellular Bgl2 produced by *Y. lipolytica* overexpressing strains

Enzyme and purification method	Total protein (mg)	Total activity (U)	Specific activity (U/mg)	Fold purification	Yield (%) recovery
Bgl1-His6					
Filtrate	169.7	543.0	–	–	100
TALON His-tag^a^	0.9	92.5	102.8	32.1	17.0
Bgl2					
Culture supernatant	2302.5	530.2	–	–	100
Ultra filtration	1986.4	510.5	–	1.1	96.3
Ion exchange	235.3	478.5	–	7.7	90.2
Gel filtration	1.8	46.4	25.8	112.2	8.8

^a^Specific activity was tested on pNP-βGlc.

Preliminary characterization of Bgl1 and Bgl2 using *p*NP-βGlc as substrate revealed that Bgl1 was 5-fold more active (102.8 U/mg) on this substrate than Bgl2 (25.8 U/mg). The activity of Bgl1 was highest at approximately pH 4.5 and 45°C, and was stable in the pH range of 4.0–5.0 and below 40°C. Regarding Bgl2, it was found to display highest activity at pH 4.0 and 50°C and was stable in the pH range of 3.5–7.0 and below 50°C (Additional file 2: Figs. S5, S6). It is noteworthy that deglycosylation of Bgl2 led to a 60% decrease in specific activity, which was probably due to its instability at 40°C (Additional file 2: Fig. S7).

### Substrate specificity and kinetic parameters of Bgl1 and Bgl2

**Fig. 4:**
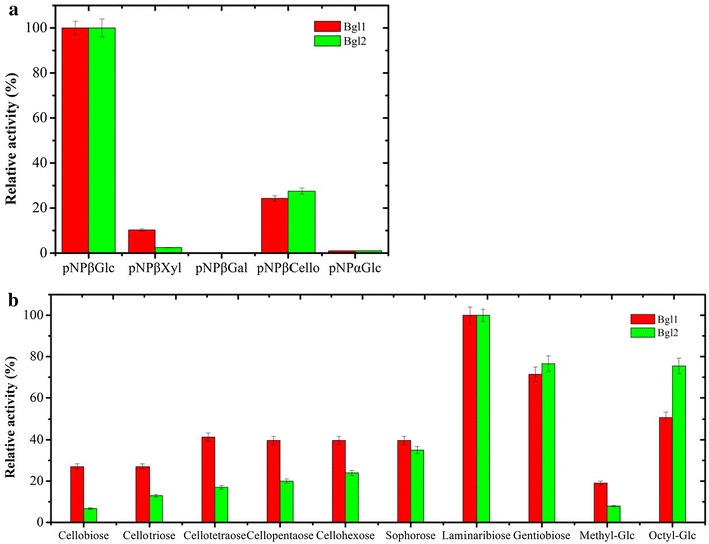
Comparison of the hydrolytic activity of β-glucosidases from *Y. lipolytica* JMY1212. Bgl1-His and Bgl2 on **a** pNP-derived substrates, and **b** natural glycosyl substrates with different β-configurations.

The substrate specificity of the purified β-glucosidases was examined using different substrates displaying α and β configurations. The results showed that both β-glucosidases were maximally active against *p*NP-βGlc (Fig. 4). However, using activity on *p*NP-βGlc as benchmark, it is noteworthy that both enzymes were active on *p*NP-β-d-cellobioside (Bgl1, 24% and Bgl2, 27%), but only Bgl1 displayed significant activity (10%) on *p*NP-β-d-xylopyranoside. Neither enzyme displayed activity on *p*NP-β-d-galactopyranoside and *p*NP-α-d-glucopyranoside.

When the activity of Bgl1 and Bgl2 on cellobiose was compared with that on other oligosaccharides, it was found that both enzymes displayed highest activity on laminaribiose (β-1, 3-linkage), followed by gentiobiose (β-1, 6-linkage), octylglucoside, sophorose (β-1, 2-linkage), cello-oligosaccharides (β-1, 4-linkage, trisaccharide and higher)) and cellobiose (β-1, 4-linkage). It is noteworthy that the hydrolytic activity of Bgl1 was less dependent on the chain length of cello-oligosaccharides, while hydrolytic activity of Bgl2 increased as the length of cello-oligosaccharides increased. Both enzymes recognized methylglucoside as substrate, but the hydrolytic activities were low compared with the other substrates (Fig. 4), indicating that correct occupation of subsite +1 is important for catalysis.

The determination of the apparent kinetic parameters of reactions catalyzed by Bgl1 and Bgl2 and containing various glucosyl disaccharides and cello-oligosaccharides revealed that the values of *K*_M_(app) and *k*_*cat*_/*K*_M_ for Bgl2-catalyzed reactions increased as a function of degree of polymerization (DP) of the cello-oligosaccharides (Table 3). In the case of Bgl1, increased DP was associated with increased *K*_M_(app) values, but not *k*_*cat*_/*K*_M_ values. Overall, considering the performance constant (*k*_*cat*_/*K*_M_), cellobiose and cellohexaose were the best substrates for Bgl1 and Bgl2, respectively. Additionally, the performance constant of Bgl1 measured on cellobiose was 12.5-fold higher than that describing Bgl2. Regarding other glucosyl substrates (i.e. those containing linkages other than β-1, 4), both Bgls displayed the highest performance constants on laminaribiose. Nevertheless, comparison of the performance constants on each of the substrates revealed that Bgl2 is less regioselective, since the *k*_*cat*_/*K*_M_ values were always lower in reactions catalyzed by Bgl1 (86% for sophorose, 47% for laminaribiose, 37% for gentiobiose, 18% for methylglucoside and 45% for octylglucoside). Finally, the lowest performance constants for both Bgls were measured for reactions containing methylglucoside.

**Table 3:** Kinetic parameters of *Y. lipolytica* Bgls for various glycoside-substrates

Substrate	Linkage	Bgl1			Bgl2		
		*K*_M_ (mM)	*k*_*cat*_ (s^−1^)	*k*_*cat*_/*K*_M_ (mM^−1^ s^−1^)	*K*_M_ (mM)	*k*_*cat*_ (s^−1^)	*k*_*cat*_/*K*_M_ (mM^−1^ s^−1^)
Cellobiose	Glc × 2, β-l, 4	0.26	21.1	81.1	0.79	5.1	6.5
Cellotriose	Glc × 3, β-1, 4	0.43	20.5	47.7	0.99	9.5	9.6
Cellotetraose	Glc × 4, β-1, 4	1.89	30.9	16.3	1.86	20.6	11
Cellopentaose	Glc × 5, β-1, 4	2.18	29.5	13.5	2.24	27.5	12.3
Cellohexaose	Glc × 6, β-1, 4	3.01	31.5	10.5	2.37	30.5	12.9
Sophorose	Glc × 2, β-1, 2	2.25	28.4	14.8	2.4	41.2	17.2
Laminaribiose	Glc × 2, β-1, 3	0.68	75.6	110.7	0.89	211.1	237.2
Gentiobiose	Glc × 2, β-1, 6	1.16	43.6	37.6	1.84	186.5	101.4
Methylglucoside	C = l	15	15	1	6.23	34.1	5.5
Octylglucoside	C = 8	0.86	32.8	38.1	1.3	111.1	85.2

The mean values of three independent experiments are shown and the standard deviation is below 10%. Hydrolytic activities for the substrate were determined from the amount of released glucose and the kinetic parameters were calculated as described in “Methods”.

### Cellobiose and cello-oligosaccharide fermentation with *Y. lipolytica* recombinant strains

**Fig. 5:**
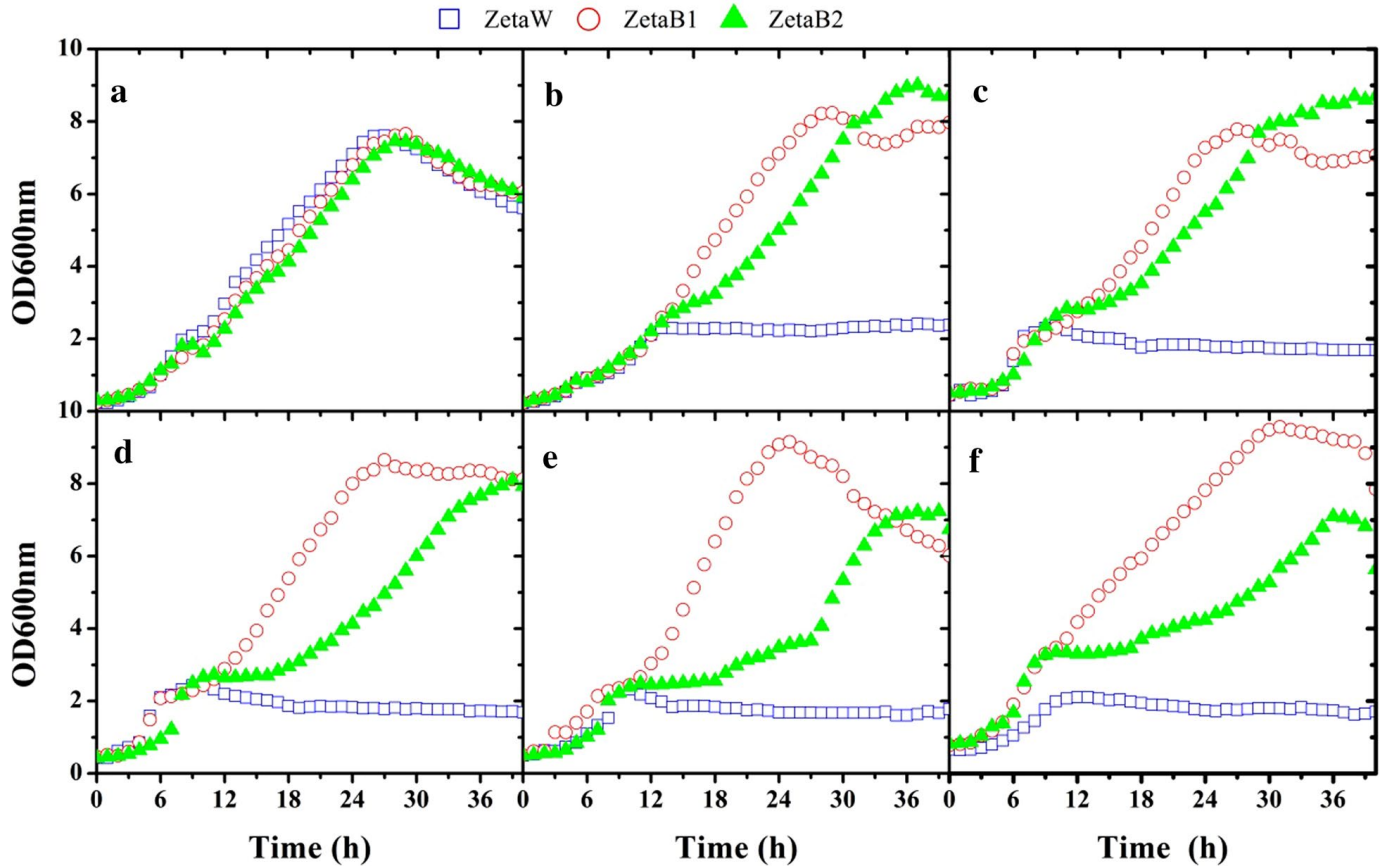
Comparison of *Y. lipolytica* ZetaW (control), ZetaB 1 (*P*_*TEF*_-*BGL1*) and ZetaB 2 (*P*_*TEF*_-*BGL2*) during aerobic growth on 5 g/L **a** glucose, **b** cellobiose, **c** cellotriose, **d** cellotetraose, **e** cellopentaose and **f** cellohexaose as carbon and energy source. Shown is OD_600nm_, optical density at 600 nm, versus time. Each data point represents the mean of at least three independent experiments and the standard deviation is less than 5%.

**Fig. 6:**
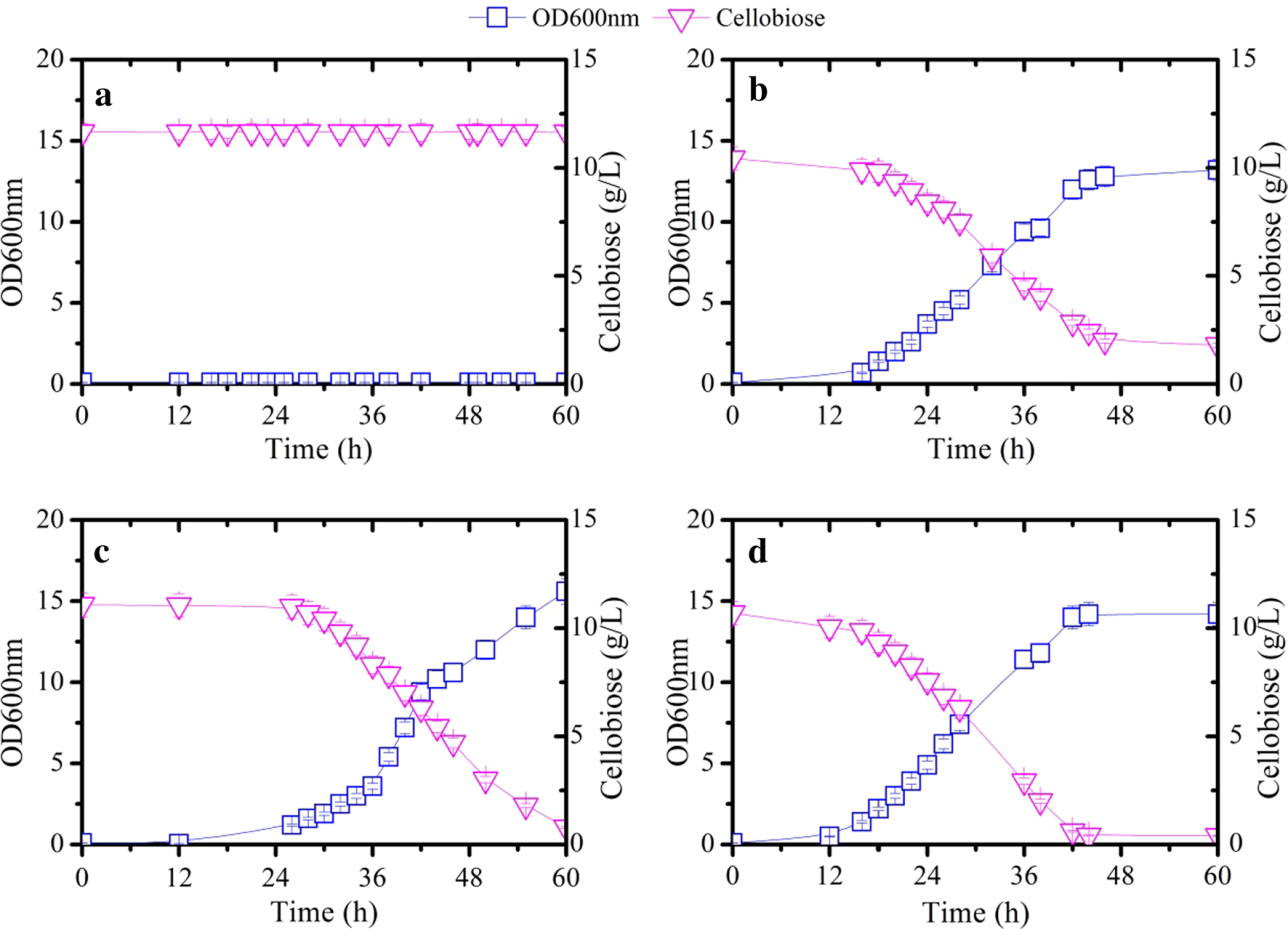
Comparison of *Y. lipolytica* **a** ZetaW (control), **b** ZetaB 1 (*P*_*TEF*_-*BGL1*), **c** ZetaB 2 (*P*_*TEF*_-*BGL2*) and **d** Zeta-B12 (*P*_*TEF*_-*BGL1*, *P*_*TEF*_-*BGL2*) during aerobic growth on 10 g/L cellobiose. Shown are OD_600nm_, optical density at 600 nm, and cellobiose concentration versus time. Each data point represents the mean of five independent experiments and the *error bar* indicates the standard deviation.

Yeast strains ZetaB 1 and ZetaB 2, expressing *BGL1* and *BGL2*, respectively, were grown in micro cultivation plates under aerobic conditions in the presence of cellobiose or cellodextrins (until Glc × 6) as sole carbon sources, using wild-type *Y. lipolytica* ZetaW as the control. The maximum specific growth rates (μ_max_) of the transformants on cellobiose were essentially the same as that of the control grown on glucose (Fig. 5a, b). ZetaB 1 grew faster than ZetaB 2 on cellobiose and cellodextrins (Fig. 5b–f), while the control was unable to grow on either of these substrates. Surprisingly, despite indications that the wild-type strain cannot grow on cellobiose or cellodextrins, the control culture (ZetaW) reached an OD value of 2.0. However, further investigation using a defined medium revealed that this unexpected growth could be attributed to the presence in the medium of 0.2% w/v casamino acids, which acted as a suitable carbon source. In defined medium, ZetaB 1 consumed 80% cellobiose over 48 h. However, upon further incubation, the remaining 20% cellobiose (at the concentration of 2 g/L) was not consumed (Fig. 6). In contrast, ZetaB 2 consumed all of the cellobiose over 60 h. Furthermore, ZetaB 1 sustained a specific growth rate (μ_max_) of 0.10 h^−1^, whereas ZetaB 2 exhibited a long lag phase on cellobiose after which two subsequent growth phases (μ_max_ values of 0.08 h^−1^ and then 0.16 h^−1^) were observed (Fig. 6; Table 4). In order to combine the advantages procured by the overexpression of *BGL1* and *BGL2* (i.e. shorter lag phase and higher cellobiose utilization, respectively), the two *BGL* sequences were cloned into JMY1212, thus yielding ZetaB 12. During cultivation on cellobiose, the performance of ZetaB 12 was the best among all the recombinant strains. It showed similar growth rate to that of the control grown on glucose and consumed 10 g/L of cellobiose within 40 h.

**Table 4:** Comparison of growth and biomass yield of *Y. lipolytica* JMY1212 control and recombinant strains in aerobic cellobiose cultivation on defined medium

Parameter	Control	ZetaB 1	ZetaB 2	ZetaB 12
μ_max_ (h^−1^) on glucose	0.15 ± 0.01	0.16 ± 0.01	0.16 ± 0.01	0.16 ± 0.01
μ_max_ (h^−1^) on cellobiose	NA	0.09 ± 0.01	0.14 ± 0.01	0.15 ± 0.01
Y_X/S_ (DCW-g/g cello)	NA	0.52 ± 0.01	0.53 ± 0.01	0.50 ± 0.01
Residue cellobiose 60 h (%)	NA	17.2 ± 1.0	7.2 ± 0.1	1.0 ± 0.3

±, the standard deviation.

*NA* not available.

### Characterization of cellulose-based lipid production by recombinant *Y. lipolytica* strains

**Fig. 7:**
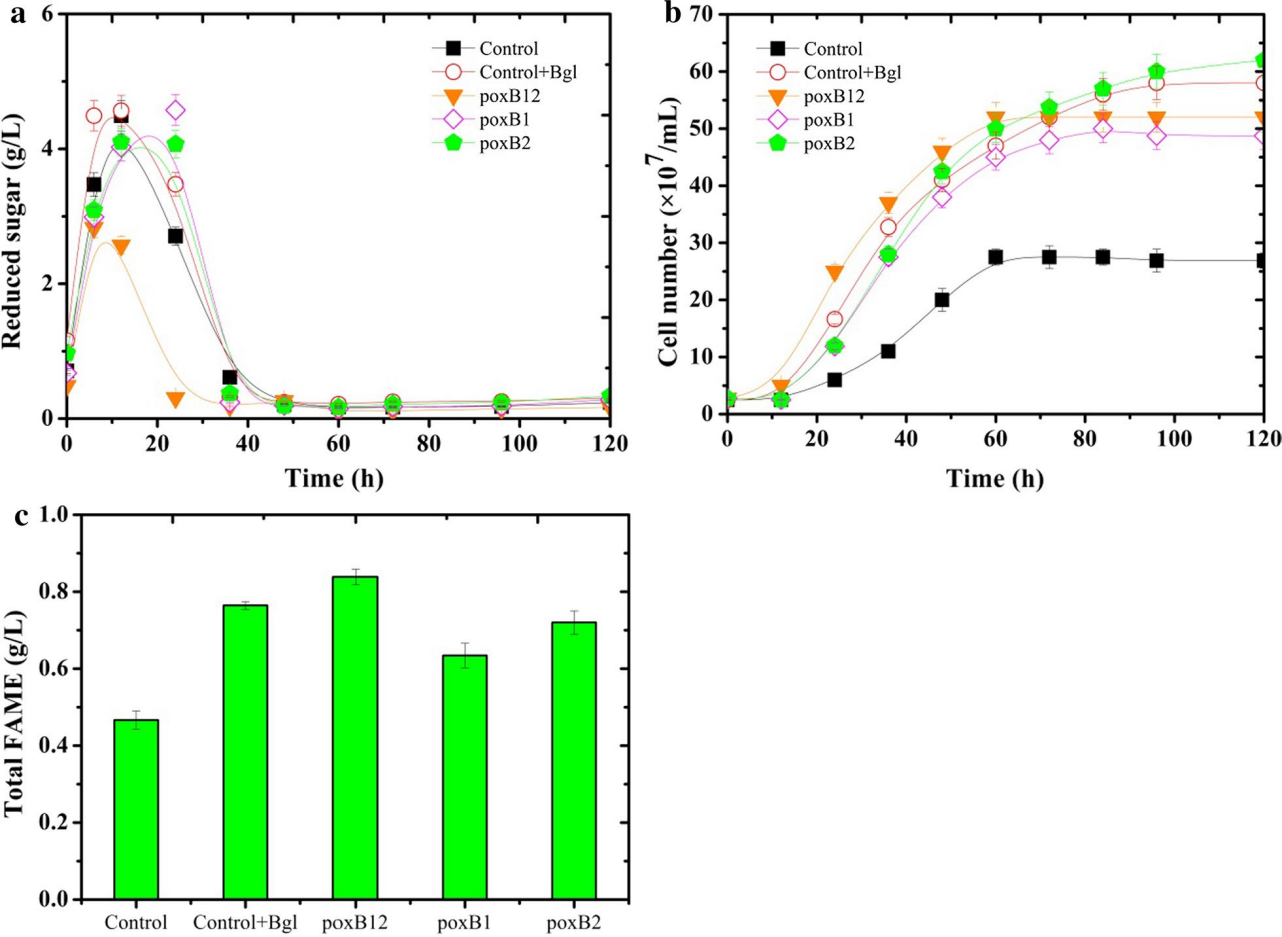
Growth and lipid production on cellulose medium of *Y. lipolytica* strains. Growth during SSF on 50 g/L cellulose supplemented with Celluclast 1.5L. **a** the concentration of reduced sugar versus time; **b** growth expressed as cell number versus time; **c** lipid content at 60 h. Strains are *Y. lipolytica* ∆*pox*B1 (*P*_*TEF*_-*BGL1*), ∆*pox*B2 (*P*_*TEF*_-*BGL2*), ∆*pox*B12 (*P*_*TEF*_-*BGL1*, *P*_*TEF*_-*BGL2*) and ∆*pox*W (wild type) under the same condition without (control) or with (control + Bgl) extra β-glucosidase (Novozyme 188). Each data point represents the mean of at least three independent experiments and the *error bars* indicate the standard deviation.

To investigate whether the *BGLs* described in this study could be used to construct a lipid-producing strain, a previously described strategy to increase lipid accumulation, involving the deletion of the 6 *POX* genes (*POX1* to *POX6*) that encode the peroxisomal acyl-coenzyme oxidases involved in lipid β-oxydation, was adopted [29]. Accordingly, *Y. lipolytica* ∆*pox*B1, ∆*pox*B2 and ∆*pox*B12 were constructed and grown on cellulose in the presence of Celluclast 1.5L. Even though this cocktail is reputedly β-glucosidase-deficient, to avoid any problems (i.e. spurious results linked to the presence of β-glucosidase in Celluclast) the Celluclast loading was kept low (7.5 FPU/g cellulose), and control experiments containing the prototrophic *Y. lipolytica* ∆*pox*W strain grown in the presence of Celluclast 1.5L with or without β-glucosidase supplementation were performed. During the initial 6 h of cultivation an accumulation of reducing sugars was observed in all of the cultures, which was attributed to Celluclast 1.5L-mediated cellulose hydrolysis. However, further monitoring revealed that after 12-h growth, less reducing sugars were present in the *Y. lipolytica* ∆*pox*B12 culture (2.7 g/L) compared to the other cultures (Fig. 7a). Moreover, this observation was correlated with continued yeast growth, whereas the growth of the other cultures stagnated over the same period (Fig. 7b). After 60 h of cultivation, the growth of ∆*pox*B12 reached a stationary phase. At this point the amount of FAMEs obtained after the methylation of Total fatty acids produced by this yeast had reached 0.8 g/L (Fig. 7c), but further growth did not result in an increase in cellular lipid content, reflecting a limitation of the available energy source. Besides the longer lag phase, the growth of ∆*pox*B1 and ∆*pox*B2 was similar to that of the control culture supplemented with β-glucosidase. Regarding the control culture, in the absence of β-glucosidase supplementation, growth ceased after 60 h and the cell density of the culture was approximately half that of the other cultures. Moreover, continuous addition of cellulases to the control culture did not procure any obvious increase in growth. When the control was supplemented with β-glucosidase, the amount of cellulose that remained unconsumed (25 g/L) was similar to that of cultures of the ∆*pox* strain expressing β-glucosidases after 5 days of growth.

## Discussion

Even though fungal β-glucosidases can be produced at relatively low cost via solid-state fermentation [30], cellulases still account for almost 50% of the cost of cellulose hydrolysis processes [31]. Moreover, taking into account the fact that the *T. reesei* secretome is rather deficient in β-glucosidases, it is particularly relevant to engineer microorganisms that are self-sufficient with regard to this type of enzyme activity.

To date, *S. cerevisiae* has been the main target for engineering aimed at the creation of a cellulolytic yeast strain for consolidated bioprocessing purposes [32, 33]. However, *Y. lipolytica* is one of the most widely studied “non conventional” oleaginous yeast species, which is well characterized for its ability to use hydrophobic substrates (e.g. alkanes, FAs, oils), glucose and glycerol as carbon sources for the production of lipids [34]. Nevertheless, *Y. lipolytica* is also known for its inability to grow on cellulose or even cellobiose. Therefore, it also constitutes an attractive target for strain engineering work.

Interestingly, comparative genomics has revealed that *Y. lipolytica* is only distantly related to the majority of yeast species and instead shares a number of common properties with filamentous fungi [35]. Therefore, based on this observation, we investigated whether *Y. lipolytica* harbors genes that allow cellobiose degradation and thus the possibility to confer cellobiose-degrading ability to *Y. lipolytica* through the use of endogenous β-glucosidases. Gratifyingly, our data clearly revealed that at least two genes, designated *BGL1* and *BGL2*, encode β-glycosidases, active under the control of TEF promoter, that hydrolyze cellobiose, although our results also indicate that these enzymes do not exclusively cleave β-1, 4 linkages. In this respect, it is noteworthy that many cellobiolytic yeasts, such as *Debaryomyces vanrijiae* [36], *Candida peltata* [37], *Monascus purpureus* [38], *Kluyveromyces fragilis* [39] and *Metschnikowia pulcherrima* [40], only produce one active Bgl and even in the case of *S. fibuligera*, which produces two active Bgls, only one of these (*Sf*Bgl1) actually hydrolyzes β-1,4 glycosidic bonds [41]. In this respect it is also intriguing to note that while Bgl1 (described herein) displays good activity on both cellobiose and cellodextrin, its homolog from *S. fibuligera* (*Sf*Bgl2, 49.5% identity) is inactive on cellobiose. Regarding the remaining four putative β-glucosidases, at this stage it is unclear why no recombinant products were revealed by western blot analysis. To pursue this further it would no doubt be useful to take a closer look at transcription, but this was not performed since the successful expression of two *BGLs* was more than enough to continue the present study.

The multiplicity of β-glucosidases in filamentous fungi is common and is often due to the presence of multiple genes, or differential post transcriptional modifications [42, 43]. However, our study provides the first evidence of possible β-glucosidase multiplicity in yeast. In filamentous fungi, the apparent redundancy of multiple cellulolytic enzymes can be explained by the ability of these microorganisms to adapt to different biomass resources and culture conditions [43] and is probably essential in fungal metabolism and survival. Similarly, in the case of *Y. lipolytica*, the presence of multiple putative β-glycosidase genes (two of which have been shown to be β-glucosidases) could be the result of adaptation of the yeast to changing environments and may help to explain the different evolutionary history of this yeast.

Considering the physicochemical characteristics of the two *Y. lipolytica* β-glucosidases described in this study, it is possible to tentatively correlate these with the cellular localization of the enzymes. Bgl2 was more stable in the assay conditions employed (50°C, pH 4.0), which might arguably be logical for an extracellular enzyme that needs to show a certain resilience to environmental challenges. On the other hand, the relative fragility of Bgl1 can be explained by the fact that its natural intracellular location probably protects it from major temperature and pH changes. In this respect the different cellular locations of the enzymes is no doubt beneficial to the yeast, since it provides optimal activity.

The comparison of the specific activities of Bgl1 and Bgl2 on cellobiose (108 units/mg and 25 units/mg protein, respectively) with that of the commercially available β-glucosidase from *Aspergillus niger* (5.2 units/mg protein), the enzyme that is generally used to complement the cellulolytic cocktail of *T. reesei* [44], is rather flattering for the former. Moreover, the *K*_M_ values describing the cellobiolytic reactions catalyzed by Bgl1 and 2 are approximately 10 and 3.4-fold lower than those of the β-glucosidases from *S. fibuligera* (2.8 mM Bgl1) and *A. niger* (2.7 mM) [44], meaning that the minimum concentration of cellobiose required for effective catalysis to occur is much lower. Likewise, comparing the apparent performance constants, *k*_cat_/*K*_M_, of *Y. lipolytica* Bgls with those of other reported β-glucosidases [36–41, 44] suggests that the enzymes described in this study hydrolyze cellodextrins more efficiently. In this respect, it is also interesting to consider the fact that Bgl1 apparently exhibits higher catalytic performance on low DP cellodextrins, while Bgl2 is more active on higher DP ones. This difference in substrate preference is consistent with the cellular location of the two enzymes (i.e. the extracellular enzymes deals with the longer oligosaccharides that cannot be transported into the cell) and possibly forms the basis of the superior performance of ZetaB 12 on Avicel compared to control cultures containing the β-glucosidase from Novozymes 188.

Apparently, in the lipid production experiments performed in this study, the activity of the *Yarrowia* β-glucosidases adequately satisfied the requirements for exo-cellulase activity, since no accumulation of reducing sugars was observed. However, lipid production was not sustained, since growth ceased before the cellulose was consumed. This failure suggests that cellulose degradation was the limiting factor, with a part of the cellulose being recalcitrant to further hydrolysis by the Celluclast cocktail, even when Bgl1 and 2 were correctly expressed and thus available.

## Conclusions

This study has provided a clear demonstration that *Y. lipolytica* does not naturally use cellobiose, despite the fact that this strain contains the genetic potential to do so. This is intriguing because the protein products of these genes are active on glucose-based oligosaccharides, including cello-oligosaccharides. Moreover, our data clearly show that upon expression of *BGL1* and *BGL2* under the control of the constitutive well characterized TEF promoter in *Y. lipolytica,* growth on cellobiose becomes possible. These encouraging findings render plausible the creation of an engineered *Y. lipolytica* strain that could be useful in advanced generation biorefinery schemes involving the use of lignocellulosic hydrolysates as feedstock for the production of bioenergy and valuable chemicals.

## Methods

### Strains and media

The genotypes of the microbial strains used in the present study are summarized in Table 5. *E. coli* DH5 were purchased from Invitrogen (Paisley, UK) and used for plasmid construction. The *Y. lipolytica* strains were routinely grown in YPD (1% yeast extract, 2% bacto peptone, and 2% glucose). Solid YPD medium contains 1.5% agar. Transformants were selected on solid YNB medium (0.17% w/v YNB, 1% glucose or cellobiose w/v, 0.5% w/v ammonium chloride, with (for Ura^+^) or without (for Leu^+^) 0.2% w/v casamino acids and 50 mM sodium–potassium phosphate buffer, pH 6.8), supplemented with uracil (440 mg/L) or leucine (440 mg/L) depending on the auxotrophic requirements. The detection of β-glucosidase activity in solid YNBcasa medium was achieved by incorporating 1.0 mM *p*-nitrophenyl-β-d-glucoside (*p*NP-βGlc) [45]. For β-glucosidase characterization, enzymes were produced in YTD medium (1% w/v yeast extract, 2% w/v tryptone, 5% w/v glucose and 100 mM phosphate buffer, pH 6.8). To compare the efficiency of recombinant β-glucosidase to degrade cellobiose and cellodextrin with respect to cell growth, yeasts were aerobically cultivated in YNBcasa medium, containing 5 g/L cellobiose or cello-oligosaccharides (C3–C6), and defined medium containing vitamins, trace elements [46] and salts, including 3.5 g/L (NH_4_)_2_SO_4_, 3.0 g/L K_2_HPO_4_, 3.0 g/L NaH_2_PO_4_ and 1.0 g/L MgSO_4_∙7H_2_O with 10 g/L cellobiose. For lipid production using cellulose as the carbon source, *Y. lipolytica* strains were grown in defined media supplemented with 50 g/L Avicel PH-101.

**Table 5:** Microbial strains used in the present study

Strains	Relevant genotype	Source of reference
*E. coli* DH5	Φ80dlacZΔm15, *recA1*, *endA1*, *gyrA96*, *thi*-*1*, *hsdR17* (rk^−^, mk^+^), *supE44*, *relA1*, *deoR*, Δ(*lacZY*A-argF) U169	Invitrogen
*Y. lipolytica* JMY1212 (Zeta)	*MATA*, *ura3*-*302*, *leu2*-*270*-*LEU2*-*zeta*, *xpr2*-*322 ∆lip2*, *∆lip7*, *∆lip8*	[50]
*Y. lipolytica* ∆*pox* JMY1233	*MATA*, *leu2*-*270*, *ura3*-*302*, *xpr2*-*322*, *pox1*-*6∆*	[29]
ZetaW	*MATA*, *xpr2*-*322*, *∆lip2*, *∆lip7*, *∆lip8*	This investigation
ZetaB 1	*P*_*TEF*_-*BGL1*	This investigation
ZetaB 2	*P*_*TEF*_-*BGL2*	This investigation
ZetaB 12	*P*_*TEF*_-*BGL1, P*_*TEF*_-*BGL2*	This investigation
∆*pox*W	*MATA*, *xpr2*-*322*, *pox1*-*6∆*	This investigation
∆*pox*B1	*P*_*TEF*_-*BGL1*	This investigation
∆*pox*B2	*P*_*TEF*_-*BGL2*	This investigation
∆*pox*B12	*P*_*TEF*_-*BGL1, P*_*TEF*_-*BGL2*	This investigation

### Plasmid constructions

The plasmids constructed in the present study are summarized in Table 6, and all primers are listed in Table 7. The plasmids used for expression of the putative β-glucosidases were constructed using the expression vectors JMP62UraTEF and JMP62LeuTEF, which are derivatives of a previously described vector [47]. Briefly, these vectors contain the *Y. lipolytica TEF* promoter and either the *URA3ex* or *LEU2ex* excisable selection markers, which are flanked by *loxP* sites and a Zeta fragment that serves as the homologous integration site [48]. Regarding β-glucosidases, six putative gene candidates (Sequences YALI0F16027g, YALI0F01672g, YALI0D18381g, YALI0B14289g, YALI0B14333g, YALI0E20185g available at Genome Resources from Yeast Chromosomes: http://gryc.inra.fr/) were identified (see Additional file 1: Table S1). For the expression of wild-type and His6-tagged proteins, the genes were amplified by PCR using FA (1–6) as forward primers and RB (1–6) or RB-His (1–6) as reverse primers, respectively. The PCR fragments were digested using either *Bam*HI/*Avr*II or *Hind*III/*Avr*II and inserted into the plasmid JMP62 UraTEF at the corresponding sites.

**Table 6:** Plasmids used or created in the present study

Plasmids	Description	Source of reference
JMP62UraTEF	*URA3*, *TEF*_*P*_-*XPR*_*T*_	[56]
JMP62LeuTEF	*LEU2*, *TEF*_*P*_-*XPR*_*T*_	[57]
JMP62UraTB1	*URA3*, *TEF*_*P*_-*BGL1*-*XPR*_*T*_	This investigation
JMP62UraTB2	*URA3*, *TEF*_*P*_-*BGL2*-*XPR*_*T*_	This investigation
JMP62LeuTB2	*LEU2*, *TEF*_*P*_-*BGL2*-*XPR*_*T*_	This investigation
JMP62UraTB12	*URA3*, *TEF*_*P*_-*BGL1*-*XPR*_*T*_, *TEF*_*P*_-*BGL2*-*XPR*_*T*_	This investigation

**Table 7:** The sequences of the oligonucleotide primers used in this study

Primer names	Sequence (5′–3′) restriction sites are italic/underlined	Restriction sites
FA1	CG^a^ GGATCCCGCGATGATCTTCTCTCTGCAACTACTAC	*Bam*HI
RB1	CGCCTAGGCTACAAAGTGAAAGTCTCACATAGC	*Avr*II
FA2	CCCAAGCTTGGGTTTGGAGGGGGTGAAAAA	*Hind*III
RB2	CCCAAGCTTGGGCTAAAGACCTAACCAATTCTTAGTCT	*Hind*III
FA3	CGGGATCCCGCGATGATTGCAAAAATACCCC	*Bam*HI
RB3	CGCCTAGGCTACTGGAGAGTAAAGGACTCG	*Avr*II
FA4	CGGGATCCCGCGATGCTCGCATTCGTCCTAC	*Bam*HI
RB4	CGGGATCCCGCTACTTGAGAGTGAAGCTGGTG	*Bam*HI
FA5	CGGGATCCCGCGATGGCTCCACCCCCGCCTCCT	*Bam*HI
RB5	CGCCTAGGTTAAGCAATCGTGATGCGACCAAGG	*Avr*II
FA6	CGCCTAGGCGCGATGGAGGAATTATCGGAGGC	*Avr*II
RB6	CGCCTAGGCTACCGGCTGAACTTCTCTTC	*Avr*II
RB-His1	^b^ *CGCCTAGGTTAATGATGGTGATGATGGTGGCTGCCGCGCGGCACCAGCCTAGG*CAAAGTGAAAGTCTCA	
RB-His2	*CCCAAGCTTGGGTTAATGATGGTGATGATGGTGGCTGCCGCGCGGCACCAGCCTAGG*AAGACCTAACCAATTCTTA	
RB-His3	*CGCCTAGGTTAATGATGGTGATGATGGTGGCTGCCGCGCGGCACCAGCCTAGG*CTGGAGAGTAAAGGA	
RB-His4	*CGGGATCCCGTTAATGATGGTGATGATGGTGGCTGCCGCGCGGCACCAGCCTAGG*CTTGAGAGTGAAGCT	
RB-His5	*CGCCTAGGTTAATGATGGTGATGATGGTGGCTGCCGCGCGGCACCAGCCTAGG*AGCAATCGTGATGC	
RB-His6	*CGCCTAGGTTAATGATGGTGATGATGGTGGCTGCCGCGCGGCACCAGCCTAGG*CTGAACTTCTCTTCC	

^a^Restriction site with corresponding restriction enzyme.

^b^His-tag introduced into the corresponding genes.

After construction, all expression vectors were verified by DNA sequencing (GATC Biotech, Konstanz, Germany). For *Y. lipolytica* transformation, vectors were digested using *Not*I, thus generating a linear DNA with Zeta sequences at both extremities, and purified. Then the linear DNA fragments were introduced into the Zeta docking platform of *Y. lipolytica* JMY1212 Zeta or randomly into the genome of ∆*pox* strain using the lithium acetate method [49]. Transformants were tested for β-glucosidase activity on YNB glucose plate containing *p*NP-βGlc and for growth on cellobiose using solid YNB cellobiose plates. Clones displaying both activities were retained for further analysis.

### Transcriptional analysis

*Y. lipolytica* JMY1212 wide type and recombinant strains overexpressing *BGL1* and *BGL2* were grown to mid-exponential phase in defined media and then transferred into fresh medium containing either glucose or cellobiose as the sole carbon source. Cells were recovered from the medium at 20 min and 1 h, respectively, and rapidly frozen in liquid nitrogen and stored at −80°C until use. Total mRNA was isolated using RNeasy Plus Mini Kit (QIAGEN) and reverse transcription was performed with iScript™ cDNA Synthesis Kit (BIO-RAD) according to the manufacturer’s instructions. Transcription of the *BGLs* was analyzed by PCR, using gene-specific primers and sequencing of the PCR products (Additional file 1: Table S3).

### Measurement of enzyme activity

β-Glucosidase activity was measured by quantifying the release of *p*NP (*p*-nitrophenol) from *p*NP-βGlc as described previously [50]. One unit of *p*NP-βGlcase activity was defined as the amount of enzyme required to release 1 μmol *p*NP per min. All protein concentrations were measured using the Bradford method and bovine serum albumin as a standard [47].

### Western blot analysis

Western blotting of proteins was performed as described previously [51]. Crude supernatant and cell-free extracts of *Y. lipolytica* JMY1212 expressing putative β-glucosidases fused with the His6 tag were concentrated 10-fold using an ultra-centrifugation filter unit (Amicon^®^ Ultra-4 10 kDa cut-off, Merk Millipore, Bedford, MA, USA). Blots were sequentially treated with mouse non position-specific His-Tag antibody 1:2,500 (THE™ from Genscript, Piscataway, NJ, USA) and the alkaline phosphatase-conjugated goat anti-mouse IgG.

### Subcellular fractionation and enzyme localization

Fractionation of yeast cells was carried out as described by Cummings and Fowler [52], with slight modifications. Briefly, yeasts were cultivated until a cellular density of 6 × 10^7^ cells/mL was reached. Then, to quantify total β-glucosidase activity, a 50-mL sample was taken and subjected to centrifugation at 8,000×*g* for 5 min at 4°C thus isolating a cell pellet and supernatant. The cell pellet was disrupted in Tris–HCl buffer (50 mM, pH 7.4, 3 mM EDTA and 0.5 mM PMSF) using a MP FastPrep-24 Instrument (MP Biomedicals Inc.). β-Glucosidase activity in both the cell lysate and the supernatant was determined as described earlier to estimate total β-glucosidase activity. Using a second 50 mL yeast culture, a cell pellet containing approximately 2 × 10^8^ cells/mL was obtained by centrifugation and then treated with zymolyase 100T at 10 mg/mL (Seikagaku corp coger) in 15 mL of sorbitol buffer (1 M sorbitol, 50 mM Tris–HCl, pH 7.4, 2 mM dithiothreitol, 10 mM MgCl_2_, 20 mM-sodium azide, 0.5 mM PMSF) at 30°C with gentle shaking. Protoplast formation was monitored using a microscope until ≥99% of the cells was lysed when SDS was added (1% SDS w/v). The solid protoplast fraction was then separated from the supernatant by centrifugation (1,000 rpm for 5 min at 4°C) and the latter was designated as the periplasmic fraction. The protoplasts were re-suspended in Tris–HCl buffer (50 mM Tris–HCl, pH 7.4) and disrupted by vortex in the presence of glass beads (0.4–0.45 mm). The homogenate was centrifuged (20,000×*g* for 2 h at 4°C) and the supernatant and solid fractions were designated as the cytoplasmic and membrane fraction, respectively. Prior to enzyme assays, the membrane fraction was suspended in citrate buffer.

### Purification of β-glucosidases

*Y. lipolytica* JMY1212 overproducing Bgl1-His6 and Bgl2 was grown in 200 mL YTD medium at 130 rpm, 28°C for 36 h before centrifugation at 8,000×*g* for 5 min. For purification of His6-Bgl1, the cell pellet was washed, suspended in 50 mL phosphate buffer (50 mM, pH 7.4) and homogenized over a 3-min period using a MP FastPrep-24 Instrument. After centrifugation (8,000×g for 5 min at 4°C), the supernatant was applied to 2 mL of TALON Metal Affinity Resin (Clontech, Takara-Bio, Kyoto, Japan) and protein was eluted using imidazole buffer according to the manufacturer’s instructions.

For purification of Bgl2, the culture supernatant was concentrated fivefold using an Amicon^®^ Ultra-4 Centrifugal Filter Unit with 30 kDa cut-off (Merk Millipore, Bedford, MA, USA). The concentrated sample was then loaded onto a Q Sepharose™ High Performance column (Hiload, 1.6 × 10 cm, Pharmacia Biotech), equilibrated with Tris-buffer (20 mM, pH8.0). The column was washed first with equilibration buffer (2 bed volumes) before applying a linear gradient of 0–1.0 M NaCl in Tris-buffer (20 mM, pH7.4) at a flow rate of 1.0 mL/min (Pharmacia Biotech ÄKTA). Eluted fractions were collected and assayed for β-glucosidase activity. All fractions displaying activity were pooled, desalted and concentrated using an Amicon ultra-filtration unit equipped with a PM-10 membrane (Millipore), before being applied to a Superdex 200 column (1.0 × 30 cm, Pharmacia Biotech) equilibrated in Tris-sodium buffer (20 mM Tris–HCl, 150 mM NaCl, pH 7.4). Protein species were separated at a flow rate of 0.5 mL/min. Fractions were collected and analyzed by SDS-PAGE to ascertain purity and estimate the approximate molecular weights of Bgl1-His6 and Bgl2. All fractions satisfying the purity criterion (>95% purity) were pooled and retained for further work.

### Deglycosylation and N-terminal amino acid sequencing

Purified Bgl1-His6 and Bgl2 were treated with endoglycosidase H (New England Biolabs, Beverly, MA, USA) according to the manufacturer’s instructions. After deglycosylation, the protein species displaying *M*_*r*_ (relative molecular mass) closest to those of the theoretical *M*_*r*_ (predicted using Protparam, http://web.expasy.org/protparam/) of Bgl1-His6 and Bgl2 were excised and submitted to N-terminal amino acid sequencing (PISSARO platform, Rouen, France).

### Physicochemical characteristics of β-glucosidases

Optimal temperatures and pH for the activity of Bgl1 and Bgl2 were determined using *p*NPGlc as the substrate. Assays were either performed at pH 5.0 and various temperatures (30–70°C) or at 30°C in variable pH conditions (2.0–8.0) using either 50 mM glycine–HCl (pH 2.0), 50 mM citrate/acetate (pH 3.0–7.2) or potassium phosphate (pH 7.0–8.2) buffer. When the temperature was varied, the pH of the citrate buffer was adjusted accordingly. Stability of Bgl1 and Bgl2 depending on pH and temperature was analyzed as follows: enzymes were incubated at 30°C for up to 2 h at various pH values (2.0–8.0) or at various temperatures (30–70°C) for up to 2 h in 50 mM citrate buffer, pH 5.0. Residual glucosidase activity was then assayed at 30°C in 50 mM citrate buffer, pH 5.0.

### Substrate specificity and enzyme kinetics

The substrate specificity of Bgl1-His6 and Bgl2 was investigated by assaying for activity on the aryl-glycosides *p*NP-β-d-glucopyranoside, *p*NP-α-d-glucopyranoside, *p*NP-β-d-galactopyranoside, pNP-β-d-xylopyranoside and pNP-β-d-cellobioside, and on the oligosaccharides cellobiose, cellotriose, cellotetraose, cellopentaose, cellohexaose, sophorose, laminaribiose, gentiobiose, methylglucoside and octylglucoside. When using aryl-substrates, the standard assay method was employed, simply replacing *p*NP-βGlc by another substrate as appropriate. For oligosaccharides, the release of glucose was quantified using an enzyme kit (d-Fructose/d-Glucose Assay Kit, liquid stable, Megazyme). To study the Michaelis–Menten parameters *K*_M_, *V*_*max*_ and *k*_*cat*_, Bgl1 (0.120 nM) or Bgl2 (0.13 nM) were added to reaction mixtures containing different substrate concentrations: 0.25–5 mM cellobiose, 0.25–5 mM cellotriose, 0.25–5 mM cellotetraose, 0.25–5 mM cellopentaose, 0.25–5 mM cellohexaose, 0.2–4 mM sophorose, 0.1–2 mM laminaribiose, 0.1–2 mM gentiobiose, 0.5–20 mM methylglucoside and 0.2–4 mM octylglucoside. Initial rates were fitted to the Michaelis–Menten kinetic equation using a nonlinear regression (SigmaPlot 10) to extract the apparent *K*_M_ and *k*_*cat*_ [53].

### Yeast growth and lipid production

Yeast growth on cellobiose and cellodextrins was performed in a 40-well microplate. A single colony from a fresh YPD plate was transferred into 5 mL of defined medium containing 10 g/L of glucose and pre-cultured until the mid-exponential phase. The cells were then harvested, washed, suspended in sterile water and used to inoculate 200 μL YNBcasa media containing 5 g/L cellobiose or cellodextrins in the microplate, achieving an initial OD_600_ of 0.1. This culture was grown in a microplate reader (Spectrostar Omega, BMG Labtech, Germany) at 30°C with continuous shaking (150 rpm) and automatic OD_600_ recording. Similarly, yeast growth on cellobiose was also performed on 30 ml defined medium containing 10 g/L cellobiose in 250 mL Erlenmeyer flasks.

For lipid production a fresh yeast culture in exponential phase was used to inoculate 50 mL defined medium containing 50 g/L Avicel in Erlenmeyer flasks, achieving an initial OD_600_ of 1.0. Celluclast 1.5L (60 FPU/mL, gift from Novozymes, Denmark) was added (7.5 U/g cellulose) and growth was pursued for 5 days (30°C, 150 rpm). Samples were taken at regular intervals to determine concentrations of biomass, glucose, cellobiose and citric acid. In parallel, two control experiments were conducted under the same conditions, with or without the addition of extra β-glucosidase (810 IU/mL Novozyme 188, gift from Novozyme, Denmark) at 12.0 IU/g cellulose as recommended [54].

### Analysis of product formation and determination of dry cell weight

To determine the concentration of substrates and extracellular metabolites, three aliquots (1.5 mL each) of cultures were rapidly frozen in liquid nitrogen and then thawed on ice before centrifugation (8,000×*g* for 5 min at 4°C) to recover supernatants for analysis. Glucose, cellobiose and citric acid were measured using an Aminex HPX87-H column (Bio-Rad Laboratories, Germany), operating at 50°C using a mobile phase (5 mM H_2_SO_4_) flowing at a rate of 0.5 mL/min. Glucose and cellobiose were detected using a Shodex RI-101 refractive index detector (Showa Denko, New York, NY, USA), while citric acid was detected using an UV detector at 210 nm (Dionex, Sunnyvale, CA, USA).

To determine the dry cell weight, three aliquots (5 mL each) of cultures were filtered through pre-weighed PES filters (0.45 μm; Sartorius Biolab, Germany). The biomass retained by the filters was washed, dried in a microwave oven at 150 W for 15 min and then placed in a desiccator before weighing. The biomass yield was calculated as the ratio of the amount of biomass obtained divided by the amount of carbon source consumed.

Lipids were extracted from freeze-dried cells (~10 mg) and methylated as described previously [55]. During the lipid extraction, C17:0 (Sigma) (50 μg) was added as the internal standard and fatty acid methyl esters (FAMEs) were analyzed by gas chromatography (6890 N Network GC System, Agilent, USA). The measurements were performed in a split mode (1 μL at 250°C), with helium as the carrier gas (2 mL/min). FAMEs were separated on a HP-5 GC column (30 m × 0.32 mm I.D., 0.5-μm film thickness, Agilent, USA). The temperature program was 120°C, ramped to 180°C (10°C/min) for 6 min, 183°C (0.33°C/min) for 9 min and 250°C (15°C/min) for 5 min. Detection was performed using a flame ionization detector (FID) at 270°C (2.0 pA). FAMEs were quantified by comparing their profiles with that of standards of known concentration.

## Additional files

Additional file 1:
**Table S1.** Six putative β-glucosidase coding genes identified harboring conserved glycosyl hydrolase family 3 N and/or 3C terminal domain. **Table S2.** The predicted N-glycosylation sites in sequence of Bgl2 by GlycoEP. **Table S3.** The sequences of the oligonucleotide primers used in verification of transcriptions. Additional file 2:
**Figure S1.** Multiple alignments of putative conserved domains of the family 3 glycosyl hydrolases of *S. fibuligera* (Bgl1, GenBank Accession numbers: AAA34314.1) against *Yarrowia* genome. **Figure S2.** Screening of *Y. lipolytica* expressing the 6 putative β-glucosidases on (a) indication plate containing YNBcasa medium supplemented with 1 mM* p*-nitrophenyl-β-D-glucoside (*p*NP-βGlc), and (b, c) YNBC plate with cellobiose as sole carbon source. **Figure S3.** Transcriptional analysis of the expression of the six putative* BGLs* in wild type strain on glucose (A) and cellobiose (B), and recombinant strain overexpression of* BGL1* and* BGL2* (C). **Figure S4.** N-terminal amino acid sequences of *Y. lipolytica* Bgl1 (A) and Bgl2 (B). The first 50 N-terminal amino acid sequences are indicated with the predicted signal sequence determined with signal P (underlined), the cleavage site predicted with a*, and the N-terminal AA sequence of the purified protein determined by direct sequencing (in bold). **Figure S5.** Optimal pH (a) and temperature (b) of Bgl1 (square) and Bgl2 (diamond) from *Y. lipolytica* JMY1212. Each data point represents the mean of three independent experiments and the error bar indicates the standard deviation. **Figure S6.** Stability of Bgl1 (a) and Bgl2 (b) from *Y. lipolytica* JMY1212 at pH from 2.0–8.0 as a function of time at 30ºC, and stability of Bgl1 (c) and Bgl2 (d) at temperature from 30ºC to 60ºC as a function of time at pH 5. Each data point represents the mean of three independent experiments and the error bar indicates the standard deviation. Please note that only one curve is given to represent the stability of Bgl2 at pH 4.0, 5.0 and 6.0 (b) and at 30ºC and 40ºC (d) as 100% of enzyme activity remained for these conditions.** Figure S7.** The hydrolytic activity of Bgl2 on* p*NP-βGlc (a) and the stability of Bgl2 at 40ºC as a function of time at pH5.0 before and after deglycosylation. Each data point represents the mean of three independent experiments and the error bar indicates the standard deviation.

## Abbreviations

LC biomass: lignocellulosic biomass
CBP: consolidated bioprocessing
FAEE: fatty acid ethyl esters
SSF: simultaneous saccharification and fermentation
FAEs: Fatty Acid Esters
DCW: dry cell weight
SCO: single cell oil
FDA: Food and Drug Administration
GRAS: generally recognized as safe
*p*NP-βGlc: *p*-nitrophenyl-β-d-glucoside
*M*_*r*_: relative molecular mass
Bgl: β-glucosidase
