# Supplementary material for: Development of cellobiose-degrading ability in Yarrowia lipolytica strain by overexpression of endogenous genes
Source: Biotechnol Biofuels. 2015 Aug 4;8:109. doi: 10.1186/s13068-015-0289-9 (PMC4524412; doi:10.1186/s13068-015-0289-9)
Supplement: Additional file 1: — Table S1. Six putative β-glucosidase coding genes identified harboring conserved glycosyl hydrolase family 3 N and/or 3C terminal domain. Table S2. The predicted N-glycosylation sites in sequence of Bgl2 by GlycoEP. Table S3. The sequences of the oligonucleotide primers used in verification of transcriptions. [file 13068_2015_289_MOESM1_ESM.docx]

Table S1 Six putative β-glucosidase coding genes identified harboring conserved glycosyl hydrolase family 3 N and/or 3C terminal domain

| GenBank Accession numbers (Protein/Gene) | The best hit by Blastp ( GenBank Accession numbers) | *Identities | **Signal Peptide |
| --- | --- | --- | --- |
| XP_500877.1/YALI0B14289g | *Saccharomycopsis fibuligera* Bgl1 (AAA34314.1) | 50.42% | Identified |
| XP_500879.1/YALI0B14333g | *Saccharomycopsis fibuligera* Bgl2 (AAA34315.1) | 45.22% | - |
| XP_502983.1/YALI0D18381g | *Talaromyces emersonii* Bgl (AAL34084.2) | 27.20% | Identified |
| XP_504177.1/YALI0E20185g | *Candida albicans* Bgl (XP_716473.1) | 26.99% | - |
| XP_504871.1/YALI0F01672g | *Kluyveromyces marxianus* Bgl (ACY95404.1) | 50.56% | - |
| XP_505480.1/YALI0F16027g | *Saccharomycopsis fibuligera* Bgl (AAA34315.1) | 49.5% | Identified |

*Identities between the YALI genes and the best hit determined by Blastp.

**Identification of signal peptide is performed by SignalP 4.1

Table S2 The predicted N-glycosylation sites in sequence of Bgl2 by GlycoEP

| Position | Residue | Score | Prediction |
| --- | --- | --- | --- |
| 60 | NMT | 0.94759377 | Potential Glycosylated |
| 68 | NIT | 0.66399635 | Potential Glycosylated |
| 260 | NGS | 1.1621016 | Potential Glycosylated |
| 331 | NGT | 0.95292889 | Potential Glycosylated |
| 361 | NFS | 1.1466943 | Potential Glycosylated |
| 367 | NDT | 1.0957472 | Potential Glycosylated |
| 382 | NVT | 1.3617419 | Potential Glycosylated |
| 421 | NPT | 0.53785594 | Potential Glycosylated |
| 484 | NIT | 0.54661313 | Potential Glycosylated |
| 496 | NLS | 0.72866348 | Potential Glycosylated |
| 534 | NLT | 0.90559006 | Potential Glycosylated |
| 575 | NVT | 0.71524064 | Potential Glycosylated |
| 614 | NDT | 1.2877947 | Potential Glycosylated |
| 665 | NIT | 1.0347556 | Potential Glycosylated |
| 705 | NES | 0.93304779 | Potential Glycosylated |
| 725 | NDT | 0.89810438 | Potential Glycosylated |
| 733 | NET | 1.062106 | Potential Glycosylated |
| 749 | NST | 1.0329935 | Potential Glycosylated |

Table S3 Sequences of the oligonucleotide primers used in verification of transcriptions

| Primer names | Sequence (5’-3’) | Target gene |
| --- | --- | --- |
| AF1 | GCGAGAAATCGTCCGAGACATCA | YALI0D08272g(*ACT1*) |
| AR2 | CAGCAGCCTCAAGACCCAGCAT |  |
| 1BF1 | AAAGGCTTCTCTACTTGAAGTGTCA | YALI0F16027g(*BGL1*) |
| 1BR2 | GACAGTCTGTCCCTGACTGGC |  |
| 2BF1 | ATATTGGAAACCTCAACATTTTCGG | YALI0B14289g(*BGL2*) |
| 2BR2 | TCAACGTTCGACAGGTTACCCC |  |
| 3BF1 | CCGACAACGAGCCAACCAAGC | YALI0D18381g(*BGL3*) |
| 3BR2 | CTGGAGCTGTTGGCAAGAGTGGTG |  |
| 4BF1 | TGGAATGACCCTTCTGGGTACTGC | YALI0F01672g(*BGL4*) |
| 4BR2 | TTGGAAACAAGTCCTCCTCGCTCT |  |
| 5BF1 | TGATGGTTTTCACTACCGGGTCG | YALI0B14333g(*BGL5*) |
| 5BR2 | GTAGGGGGTCACAACGAACGGA |  |
| 6BF1 | GCGTCCTCCAACCATACATCGG | YALI0E20185g(*BGL6*) |
| 6BR2 | CGTTTCCACCATCCACGTCTTTC |  |
