# Supplementary material for: Development of cellobiose-degrading ability in Yarrowia lipolytica strain by overexpression of endogenous genes
Source: Biotechnol Biofuels. 2015 Aug 4;8:109. doi: 10.1186/s13068-015-0289-9 (PMC4524412; doi:10.1186/s13068-015-0289-9)
Supplement: Additional file 2: — Figure S1. Multiple alignments of putative conserved domains of the family 3 glycosyl hydrolases of S. fibuligera (Bgl1, GenBank Accession numbers: AAA34314.1) against Yarrowia genome. Figure S2. Screening of Y. lipolytica expressing the 6 putative β-glucosidases on (a) indication plate containing YNBcasa medium supplemented with 1 mM p-nitrophenyl-β-D-glucoside (pNP-βGlc), and (b, c) YNBC plate with cellobiose as sole carbon source. Figure S3. Transcriptional analysis of the expression of the six putative BGLs in wild type strain on glucose (A) and cellobiose (B), and recombinant strain overexpression of BGL1 and BGL2 (C). Figure S4. N-terminal amino acid sequences of Y. lipolytica Bgl1 (A) and Bgl2 (B). The first 50 N-terminal amino acid sequences are indicated with the predicted signal sequence determined with signal P (underlined), the cleavage site predicted with a*, and the N-terminal AA sequence of the purified protein determined by direct sequencing (in bold). Figure S5. Optimal pH (a) and temperature (b) of Bgl1 (square) and Bgl2 (diamond) from Y. lipolytica JMY1212. Each data point represents the mean of three independent experiments and the error bar indicates the standard deviation. Figure S6. Stability of Bgl1 (a) and Bgl2 (b) from Y. lipolytica JMY1212 at pH from 2.0–8.0 as a function of time at 30ºC, and stability of Bgl1 (c) and Bgl2 (d) at temperature from 30ºC to 60ºC as a function of time at pH 5. Each data point represents the mean of three independent experiments and the error bar indicates the standard deviation. Please note that only one curve is given to represent the stability of Bgl2 at pH 4.0, 5.0 and 6.0 (b) and at 30ºC and 40ºC (d) as 100% of enzyme activity remained for these conditions. Figure S7. The hydrolytic activity of Bgl2 on pNP-βGlc (a) and the stability of Bgl2 at 40ºC as a function of time at pH5.0 before and after deglycosylation. Each data point represents the mean of three independent experiments and the error bar indicate [file 13068_2015_289_MOESM2_ESM.docx]

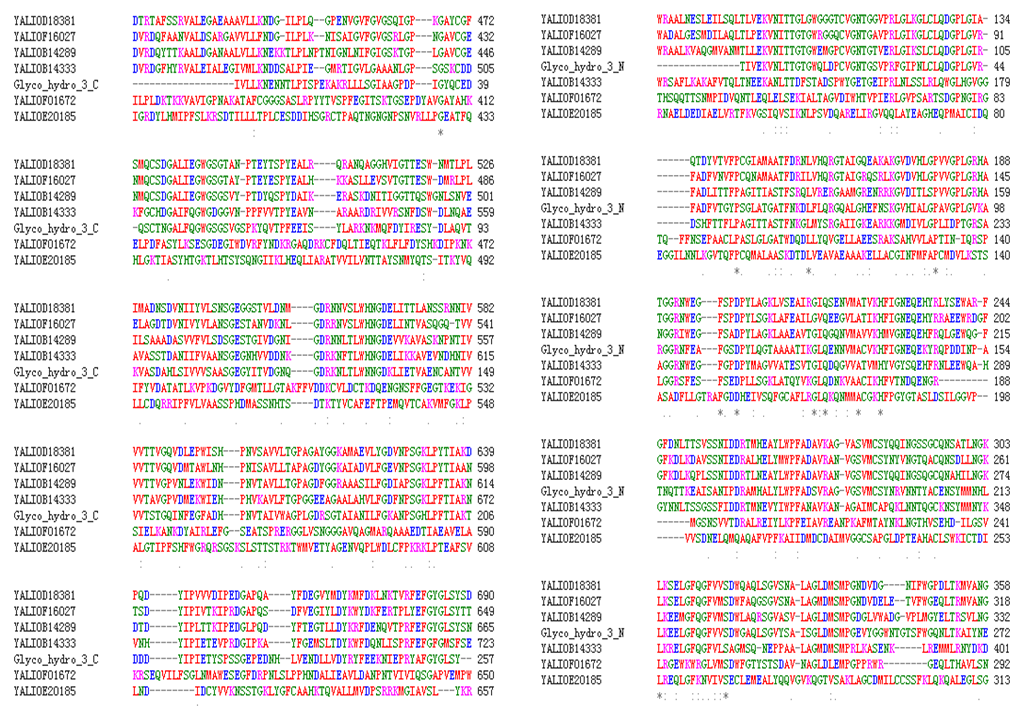


Figure S1 multiple alignments of putative conserved domains of the family 3 glycosyl hydrolases of *S. fibuligera* (Bgl1, GenBank Accession numbers: AAA34314.1) against *Yarrowia* genome.


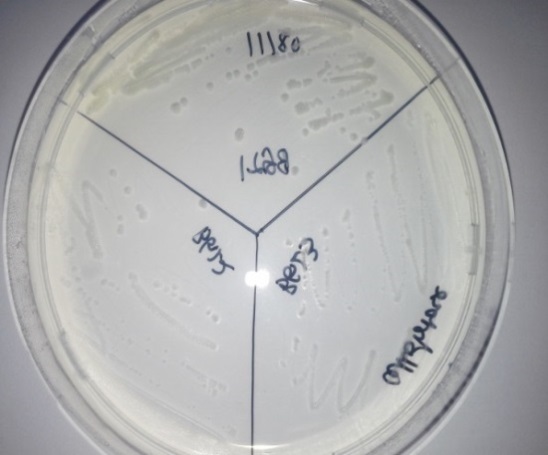


**b**

ZetaB1

ZetaB4

ZetaB3


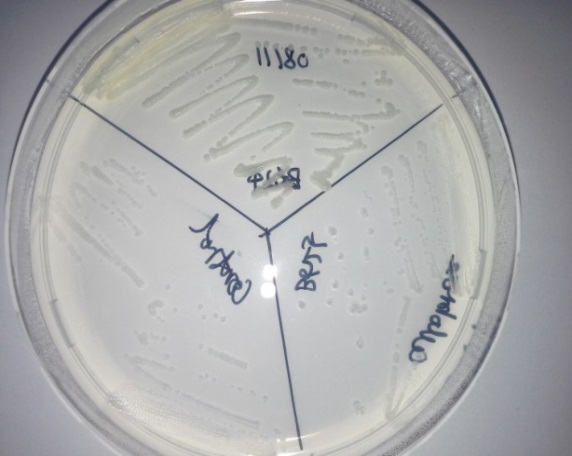


**c**

ZetaB2

Zeta-Control

ZetaB5

Zeta-B6


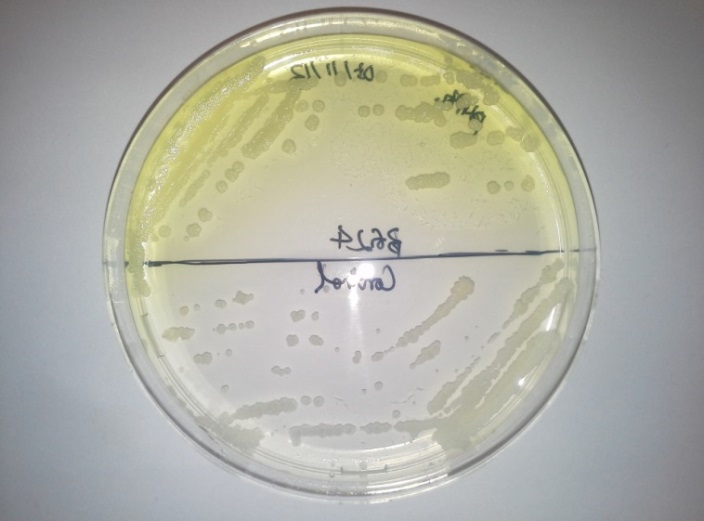


**a**

Zeta-Control

ZetaB2

Figure S2 Screening of *Y. lipolytica* expressing the 6 putative β-glucosidases on (a) indication plate containing YNBcasa medium supplemented with 1 mM *p*-nitrophenyl-β-D-glucoside (*p*NP-βGlc), and (b, c) YNBC plate with cellobiose as sole carbon source.

Figure S3 Transcriptional analysis of the expression of the six putative *BGLs* in wild type strain on glucose (A) and cellobiose (B), and recombinant strain overexpression of *BGL1* and *BGL2* (C).


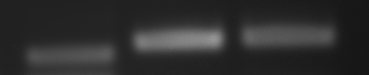

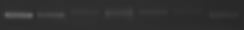

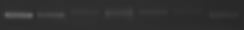


A *ACT1*  *BGL1 BGL2 BGL3 BGL4 BGL5 BGL6*

C *ACT1*  *BGL1 BGL2*

B *ACT1*  *BGL1 BGL2 BGL3 BGL4 BGL5 BGL6*

A

MIFSLQLLLTTALAAS***SPDWYPTPEI**GSITSDWADALGESMDILAQLTLP

1 17

B

MLAFVLLLTMLLAAALA***DPFSDKDAY**KHSPPYYPAPEIGRVPTDLRWRAA

1 18

Figure S4 N-terminal amino acid sequences of *Y. lipolytica* Bgl1 (A) and Bgl2 (B). The first 50 N-terminal amino acid sequences are indicated with the predicted signal sequence determined with signal P (underlined), the cleavage site predicted with a *, and the N-terminal AA sequence of the purified protein determined by direct sequencing (in bold).

Figure S5 Optimal pH (a) and temperature (b) of Bgl1 (square) and Bgl2 (diamond) from *Y. lipolytica* JMY1212. Each data point represents the mean of three independent experiments and the error bar indicates the standard deviation.

Figure S6 Stability of Bgl1 (a) and Bgl2 (b) from *Y. lipolytica* JMY1212 at pH from 2.0-8.0 as a function of time at 30ºC, and stability of Bgl1 (c) and Bgl2 (d) at temperature from 30ºC to 60ºC as a function of time at pH 5. Each data point represents the mean of three independent experiments and the error bar indicates the standard deviation. Please note that only one curve is given to represent the stability of Bgl2 at pH 4.0, 5.0 and 6.0 (b) and at 30 ºC and 40ºC (d) as 100% of enzyme activity remained for these conditions.

Figure S7 The hydrolytic activity of Bgl2 on *p*NP-βGlc (a) and the stability of Bgl2 at 40ºC as a function of time at pH5.0 before and after deglycosylation. Each data point represents the mean of three independent experiments and the error bar indicates the standard deviation.
